# Spectral CT for preoperative prediction of lymphovascular invasion in resectable gastric cancer: With external prospective validation

**DOI:** 10.3389/fonc.2022.942425

**Published:** 2022-10-04

**Authors:** Jing Li, Yi Wang, Rui Wang, Jian-bo Gao, Jin-rong Qu

**Affiliations:** ^1^ Department of Radiology, The Affiliated Cancer Hospital of Zhengzhou University (Henan Cancer Hospital), Zhengzhou, China; ^2^ Department of Pathology, The Affiliated Cancer Hospital of Zhengzhou University (Henan Cancer Hospital), Zhengzhou, China; ^3^ Department of Radiology, The first Affiliated Hospital of Zhengzhou University, Zhengzhou, China

**Keywords:** spectral CT, iodine concentration, lymphovascular invasion, gastric cancer, nomogram

## Abstract

**Objectives:**

To develop and externally validate a spectral CT based nomogram for the preoperative prediction of LVI in patients with resectable GC.

**Methods:**

The two centered study contained a retrospective primary dataset of 224 pathologically confirmed gastric adenocarcinomas (161 males, 63 females; mean age: 60.57 ± 10.81 years, range: 20-86 years) and an external prospective validation dataset from the second hospital (77 males and 35 females; mean age, 61.05 ± 10.51 years, range, 31 to 86 years). Triple-phase enhanced CT scans with gemstone spectral imaging mode were performed within one week before surgery. The clinicopathological characteristics were collected, the iodine concentration (IC) of the primary tumours at arterial phase (AP), venous phase (VP), and delayed phase (DP) were measured and then normalized to aorta (nICs). Univariable analysis was used to compare the differences of clinicopathological and IC values between LVI positive and negative groups. Independent predictors for LVI were screened by multivariable logistic regression analysis in primary dataset and used to develop a nomogram, and its performance was evaluated by using ROC analysis and tested in validation dataset. Its clinical use was evaluated by decision curve analysis (DCA).

**Results:**

Tumor thickness, Borrmann classification, CT reported lymph node (LN) status and nICDP were independent predictors for LVI, and the nomogram based on these indicators was significantly associated with LVI (*P*<0.001). It yielded an AUC of 0.825 (95% confidence interval [95% CI], 0.769-0.872) and 0.802 (95% CI, 0.716-0.871) in primary and validation datasets (all *P*<0.05), with promising clinical utility by DCA.

**Conclusion:**

This study presented a dual energy CT quantification based nomogram, which enables preferable preoperative individualized prediction of LVI in patients with GC.

## Highlights

- This study firstly developed and externally validated a dual-energy CT based nomogram to predict lymphovascular invasion in patients with resectable gastric cancer.
- The nomogram incorporated risk factors of tumor thickness, Borrmann classification, CT reported LN status and normalized iodine concentration at delay phase, which enable superior preoperative individualized prediction of lymphovascular invasion in gastric cancer.
- Normalized iodine concentration at delayed phase was an independent predictor for lymphovascular invasion, which indicates the importance of delayed enhanced scan in quantitative description of aggressiveness in gastric cancer.

## Introduction

Gastric cancer (GC) is the fifth most common cancer and the third leading cause of cancer related deaths worldwide, despite declining morbidity and mortality in the past five years (1). Curative surgery is the best treatment option for patients with resectable advanced GC, but with local recurrence up to 30% of patients (2). Although the American Joint Committee on Cancer (AJCC) was considered the gold standard to predict outcome generally, it failed to predict heterogeneous survival rates individually in GC patients with the same stage (3, 4). Recently, studies have revealed that lymphovascular invasion (LVI) was associated with recurrence and prognosis in GC patients (5, 6), patients in LVI positive status after surgery presented with higher possibility of recurrence and poorer 5 years’ survival. Thus, some researchers (7, 8) recommended combining LVI in risk stratification of prognosis and selection criteria for the need of adjuvant therapies to improve overall survival in GC patients.

LVI refers to tumor cells invading into lymphatic and/or blood vessel near tumor, and serves as an important path of locoregional tumor dissemination (9, 10), and is a predictor of lymph node metastasis (LNM) and biological aggressiveness in GC (11). Despite the significant prognostic importance of LVI, it only can be acquired on surgical specimen, this hysteretic nature limits its use in preoperative practice stage. Therefore, finding a preoperative maker to predict LVI status is clinically desirable. Meng Y et al. (12) have developed a nomogram based on pre-operative features to predict LVI, but without involving quantitative indicators on enhanced CT. Ma Z et al. (13) found correlation of LVI with CT attenuation values on multiphasic enhanced CT, but without external validation of the results. To date, some researchers have focus on the prediction of LVI using the emerging radiomics and deep learning algorithm (14, 15), but these single center based radiomics have limitations for being accepted as broad consensus classifier due to the lack of simplicity, reproducibility, repeatability, and availability in real practice.

Spectral CT is the milestone in the development of CT technique, has greatly improved the diagnostic ability in tumor staging and therapeutic efficacy evaluation for GC (16, 17). Previous study have revealed ICs derived from spectral CT are associated with angiogenesis in GC (18), tumor angiogenesis is highly related to LVI in patients with GC (19, 20), and a groundwork have proved IC in venous phase is a promising predictor for LNM in GC (21). Thus, we hypothesis that the incorporation of quantitative dual energy data could further improve the preoperative prediction of LVI in GC. To our knowledge, there is no research on the relationship between spectral CT and LVI is GC. Therefore, the aim of the study is to investigate the predictive value of spectral CT quantification for LVI in GC, by primarily developing an IC based nomogram in a retrospective cohort, then validating its efficacy in a prospective cohort externally.

## Material and methods

### Patients

**Figure 1:**
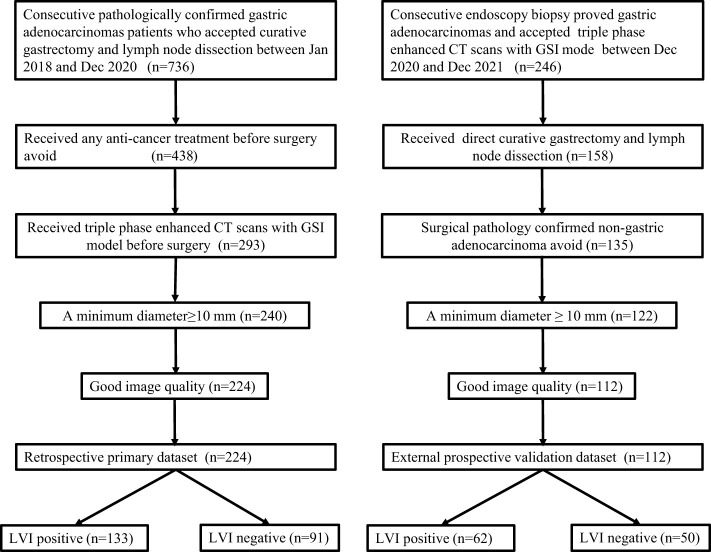
Flowchart of patient’s enrollment in the primary and validation dataset.

The institutional review board approved this study. The requirement for informed consent was waived in the primary dataset because the retrospective nature, and was obtained from each patient in the prospective validation dataset (NCT04028375). The primary dataset comprised an evaluation of imaging data and medical records between Jan 2018 and Dec 2020 to identify patients with histologically confirmed gastric adenocarcinomas who underwent surgical resection with curative intent. The validation dataset consisted of patients with histologically confirmed GC who underwent surgical resection and gemstone spectral imaging (GSI) enhanced scans before surgery between Dec 2020 and Dec 2021. The inclusion criteria, exclusion criteria and recruitment pathway of patients were presented in **Figure 1**. In total, 224 consecutive patients were identified and comprised the primary dataset: 161 males and 63 females, mean age, 60.33 ± 11.19 years, range20-85 years. An independent external validation dataset of 112 consecutive patients (77 males and 35 females; mean age, 61.05 ± 10.51 years, range, 31 to 86 years) was selected from 246 consecutive patients according to the inclusion criteria and exclusion criteria presented in **Figure 1**. Clinical data, including age and gender, tumor location was obtained from medical records.

### CT imaging study

CT data in the primary dataset were collected on two spectral CT scanner with GSI mode (Discovery CT scanner and Revolution CT scanner, both from GE Medical System). Prospective CT data in the external validation dataset were acquired on Revolution CT scanner. All patients were overnight fasted, 20 mg of scopolamine (Hangzhou Minsheng Pharmaceutical Group Co., Ltd. Specifications: 10 mg/mL) were administered intramuscularly to reduce gastrointestinal peristalsis 20 min before CT examination. Patients drank 600-1000 ml warm water to distend the stomach prior to CT examination. The CT scans, covering the entire stomach region, were acquired with breath-hold with the patient supine in all of the phases. For enhanced CT scans, patients were infused 1.5 ml/kg of ionic contrast agent (Ultravist 370, Bayer Schering Pharma) with a pump injector (Urich REF XD 2060-Touch, Ulrich Medical) at a rate of 3.0 ml/s into the antecubital vein. Arterial phase (AP), venous phase (VP) and delayed phase (DP) contrast enhanced CT images were established at 30s, 60 s and 90s after contrast agent injection. The other acquisition parameters were as follows: (1) tube voltage of spectral imaging mode switching between 80 kVp and 140 kVp; (2) tube current 375 mA on Discovery CT scanner (selected optimization), and 400 mA on Revolution CT; (3) rotation time of 0.8s; (4) detector collimation of 64×0.625mm; (5) image matrix of 512×512; (6) FOV (field of view) of 380 mm×380 mm, 400 mm×380 mm; (8) reconstruction section thickness of 1.25 mm; (9) pitch of 1.375:1 for Discovery CT scanner, 0.992:1 for Revolution CT. An adaptive statistical iterative reconstruction (ASIR, index ¼ 30%) and adaptive statistical iterative reconstruction veo (ASIR-V, index ¼ 50%) algorithm was used on Discovery CT and Revolution CT platform respectively to reduce image noise and the radiation dose on spectral CT.

### Image interpretation

The CT images were transferred to GE ADW 4.7 workstation (GE Healthcare, Milwaukee, WI, USA) and interpreted by two radiologists (6 and 10 years of experience in gastrointestinal radiology) on GSI Viewer software with a standard soft-tissue window (Window Level 40 and Window Width 400). Before analysis, the two radiologists were informed of tumor location. Because tumor thickness was proved to be a risk factor for LNM in a prior study (21), it was included in this present study continuously, defined as the maximal diameter perpendicular to the longest axis on the maximal cross-section. Borrmann classification on CT was evaluated according to tumor morphology, infiltration scale and presence of ulceration (21, 22). Circumscribed mass as classification I, circumscribed mass presented with ulcers as II, infiltrative mass with ulcers as III, and diffuse infiltrative mass as IV (16). Clinical T staging (cT) was evaluated by the invasion depth of tumor; CT reported LN status, the presence of either regional LN of >10 mm with or without heterogeneous enhancement and/or clusters of ≥3 lymph nodes was scored as CT reported LN positive, and vice versa (17). All the imaging features was evaluated by reaching two readers’ consistency, if there was divergence between the two readers for classification of any features, a third senior reader was included for reaching a consensus or obeying the majoritarian.

ICs in the arterial phase (ICAP), venous phase (ICVP) and delayed phase (ICDP) were measured separately. A free hand, phase-based individualized ROI outlining the whole tumor profile was manually drawn on material deposition (MD) images in the largest cross-sectional area by the two radiologists independently, and then the IC value was automatically generated. ICs of the aorta were obtained by placing circular ROIs at the same slice, avoiding calcified plaque. Then IC in the tumor was normalized by dividing IC of tumor to that of aorta to derive a normalized iodine concentration (nIC=IClesion/ICaorta) (21). All measurements were repeated three times, and the average values were calculated.

### Histopathology

Samples were obtained from each surgical specimen, and pathologic indicators was analyzed using hematoxylin and eosin stained 4um thick sections. Each slide was independently analyzed by two experienced pathologists who were masked to imaging findings. Consensus was reached by discussion or introduction of a third pathologist for uncertain cases. LVI was defined as the presence of tumor emboli within either the lymphatic or vascular channels (9, 10). The other pathologic parameters included: T staging, N staging, perineural invasion, histodifferentiation, ulceration, Lauren subtype, positive node numbers (PN), total dissected node numbers (TN), and positive lymph node ratios (PNR) was also recorded. TN was defined as the total number of dissected nodes, PN was defined as number of pathologically diagnosed metastatic nodes, PNR is the ratio of PN to TN (PNR=PN/TN).

### Statistical analyses

Statistical analyses were performed using SPSS software (version 23.0), MedCalc software (version 18.0) and R software (version 3.6.1). Interobserver agreements were assessed by intraclass correlation coefficient (ICC). Kolmogorov-Smirnov test was used to check the normality assumption. Enumeration data were compared *via* Student’s *t* test or Mann-Whitney U test. Categorical data were compared through chi-square test or Fisher’ exact test. Beginning with the significant variables in the unviable analysis, multivariable logistic regression with backward step wise selection was applied to identify independent predictors based on the primary cohort. Using the regression coefficients, an easy-to-use nomogram was built to predict the individual probability of LVI. The predictive value of nomogram was assessed with the area under curve (AUC) of the receiver operating characteristic (ROC) curve with 95% confidence intervals (95% CIs). The differences of AUCs among nomogram and ICs in each dataset, as well as AUCs yielded by the nomogram between the primary and validation datasets were compared by Delong test. The calibration curve and decision curve were plotted using the “rms” package (version 6.2) and the “rmda” package (version 1.6), respectively. A two-sided p value less than 0.05 was considered statistically significant.

## Results

### Interobserver agreement

The interobserver agreement between two readers was excellent, and the ICC value was 0.955, 0.976, 0.934, 0.912, 0.943, 0.925 respectively, for ICAP, ICVP, ICDP, nICAP, nICVP and ICDP measurements.

### Demographic and pathological characteristics

A total of 336 GC patients (238 males, 98 females; mean age: 60.57 ± 10.96 years, range: 20-86 years) were included. Tumor thickness range from 5.3 to 38.2 mm, (mean: 14.90 ± 5.89 mm). Patient characteristics in the primary and validation cohorts were listed in **Table 1**. There were no significant differences between the two cohorts in LVI positive prevalence (59.38% in primary cohort and 55.46% in validation cohort (*χ^2 =^* 0.495, *P=*0.278) and other background clinicopathological characteristics. There were no significant differences in clinical characteristics between the primary and the validation dataset neither within the LVI positive cohort (*t*=1.686, *P* = 0.684 for age; *χ^2 =^* 2.347, *P*=0.556 for sex; *χ^2 =^* 4.403, *P*=0.221 for location) nor the LVI negative cohort (*t*=1.125, *P*= 0.263 for age, *χ^2 =^* 2.693, *P*=0.117 for sex; *χ^2 =^* 2.657, *P*=0.448 for location). **Table 2** illustrated the comparison of clinicopathological characteristics between LVI (+) and LVI (-) groups in both primary dataset and validation dataset. Except tumor location, age and gender, the other clinicopathologic characteristics were statistically different between LVI positive and negative groups in both primary and validation dataset, justifying their use as training and validation datasets. LVI positive group contained more patients with T3-4a, LNM, poor differentiated, positive LVI, greater TN, PN, PNR, but less patients in intestinal Lauren subtype in both primary and validation datasets.

**Table 1:** Patients background characteristics between primary dataset and validation datasets.

Characteristics		Primary dataset	Validation dataset	*t/Z/χ^2^*	*P*
Age	Range: 24-85	60.33 ± 11.19	61.05 ± 10.51	-0.573	0.567
Sex	Male	161	77	0.353	0.532
	Female	63	35		
LVI	Positive	133	62	0.495	0.278
	Negative	91	50		
Tumor location	Cardia/Fundus	78	51	6.656	0.084
	Body	68	36		
	Antrum	76	25		
	≥2/3 stomach	2	0		
pT	1	56	22	3.912	0.271
	2	46	27		
	3	77	46		
	4a	45	16		
pN	0	100	49	1.622	0.805
	1	37	21		
	2	48	26		
	3a	30	10		
	3b	9	6		
PN*	Range:0-28	1 (0-5)	1 (0-4)	0.545	0.586
TN*	Range:0-93.33	25 (19.25-31)	25 (19-33)	0.835	0.404
PNR*(%)	Range:0-78	5.26 (0-20)	2.54 (0-17.21)	0.567	0.572
Differentiation	Good	128	54	5.271	0.072
	Moderate	83	55		
	Poor	13	3		
Ulceration	Present	162	83	0.137	0.795
	Absent	62	29		
Lauren subtype	Intestinal	62	29	2.100	0.552
	Mixed	79	34		
	Diffused	83	49		
Perineural invasion	Negative	113	56	0.006	0.939
	Positive	111	56		

*PN, positive node numbers; TN, total dissected nodes numbers; PNR, positive node ratio=PN/TN (%). Comparison of PN, TN, PNR between two datasets using Mann-Whitney U or Wilcoxon W test. The median value (25%quanter, 75%quanter) of PN, TN, PNR in primary dataset was 1(0-5), 25 (19.25-31), 5.26(0-20) with range of 0-28, 0-78, 0-90.91%, respectively; and was 1 (0-4), 25 (19-33), 2.54(0-17.21) in validation dataset, with range of 0-25, 0-70, 0-86.21%, respectively.

**Table 2:** Comparison of clinicopathological characteristics between LVI positive and LVI negative groups in the primary and validation datasets.

Characteristics		Primary dataset				Validation dataset			
		LVI (-)(n = 91)	LVI (+)(n = 133)	*t/Z/χ^2^*	*P*	LVI (-)(n = 50)	LVI (+)(n = 62)	*t/Z/χ^2^*	*P*
Age mean± SD, years	Range: 24-85	61.18 ± 10.46	59.74 ± 11.68	0.940	0.348	59.08 ± 10.82	62.65 ± 10.06	1.803	0.052
Sex				1.932	0.177			0.949	0.413
	Male	70	91			32	45		
	Female	21	42			18	17		
Tumor location				0.255	0.968			10.710	0.004
	Cardia/Fundus	31	47			19	10		
	Body	29	39			27	35		
	Antrum	30	46			4	17		
	≥2/3 stomach	1	1			0	0		
pT	1	50	6	87.073	<0.001	18	4	34.259	<0.001
	2	22	24			19	8		
	3	12	65			10	37		
	4a	7	38			3	13		
pN	0	85	15	147.899	<0.001	43	6	68.080	<0.001
	1	1	36			3	18		
	2	4	44			2	24		
	3a	1	29			1	9		
	3b	0	9			0	5		
PN*	Range:0-28	0 (0, 0)	4 (1.5, 8)	11.243	<0.001	0 (0,0)	4 (1, 6)	8.010	<0.001
TN*	Range:0-93.33%	22 (19, 30)	25 (20, 31)	1.627	0.052	25 (19.75,30.25)	26 (19,37.25)	6.712	0.479
PNR*(%)	Range:0-78	0 (0, 0)	15.00 (5.72,33.60)	10.854	<0.001	0 (0, 0)	13.9 (5.80,28.36)	7.984	<0.001
Differentiation	Good	10	3	10.640	0.005	3	0	4.597	0.100
	Moderate	38	45			26	29		
	Poor	43	85			21	33		
Ulceration	Present	46	116	37.041	<0.001	31	52	6.900	0.010
	Absent	45	17			19	10		
Lauren subtype	Intestinal	33	29	9.043	0.024	17	12	3.141	0.208
	Mixed	31	49			14	20		
	Diffused	27	55			19	30		
Perineural invasion	Negative	73	38	57.657	<0.001	35	21	14.452	<0.001
	Positive	18	95			15	41		

LVI, perineural invasion; (-), negative; (+), positive. *PN, positive node numbers; TN, total dissected nodes numbers; PNR, positive node ratio=PN/TN (%). Comparison of PN, TN, PNR between two groups using Mann-Whitney U or Wilcoxon W test. The median value (25%quanter, 75%quanter) of PN, TN, PNR in primary dataset was 1(0-5), 25 (19.25-31), 5.26(0-20) with range of 0-28, 0-78, 0-90.91%, respectively; and was 1 (0-4), 25 (19-33), 2.54(0-17.21) in validation dataset, with range of 0-25, 0-70, 0-86.21%, respectively. The mean value here is the mean rank calculated by statistical analysis.

### CT imaging features

**Figure 2:**
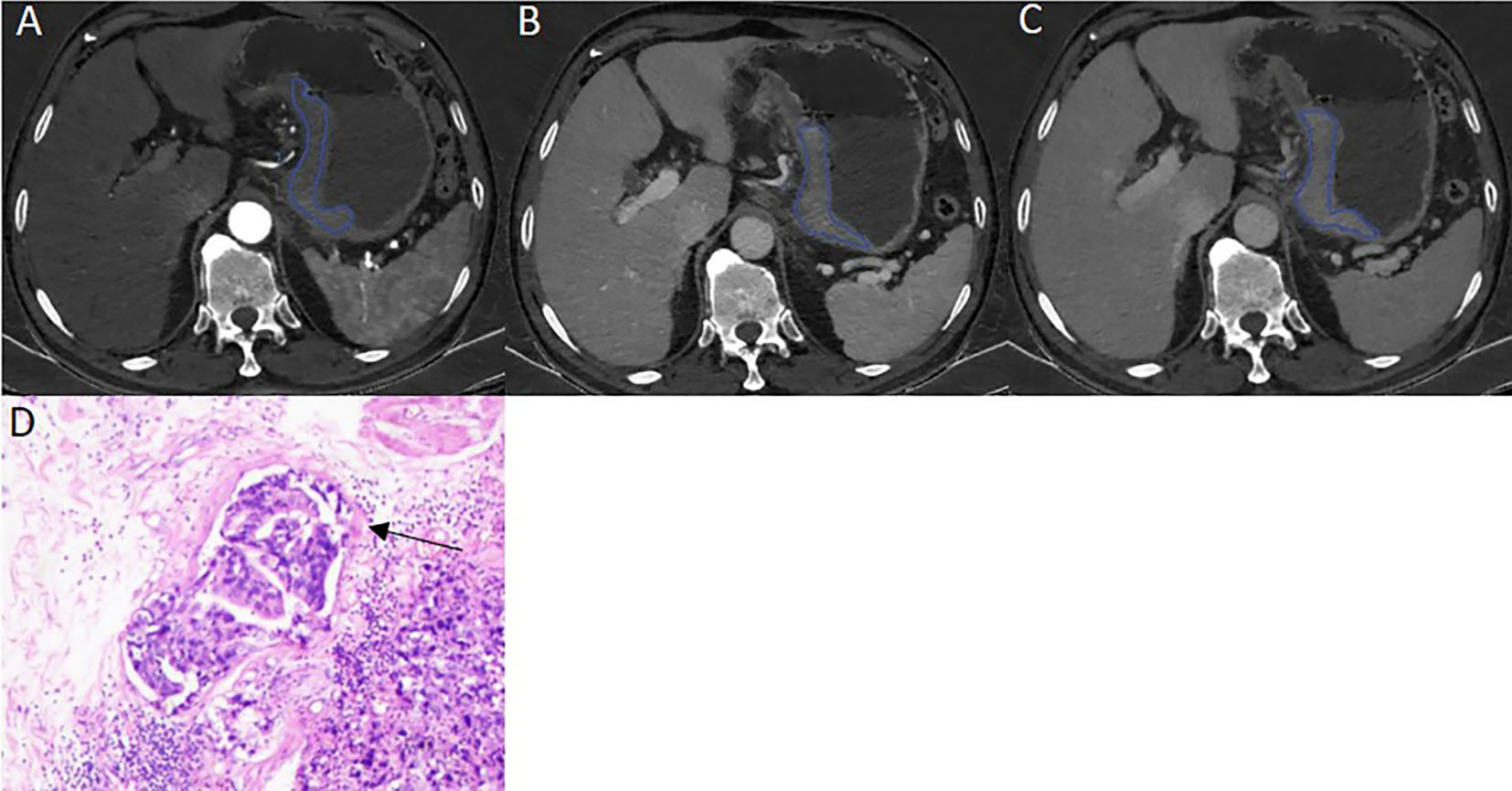
A 57 years old male patient with pathologically confirmed gastric adenocarcinoma, staging of pT4aN3aM0, LVI positive. The tumor thickness was 21.72mm, Borrmann classification of III. **(A)** Iodine map at arterial phase, tumor was hyperintense, IC value was 23.11 (100μg/ml); **(B)** Iodine map at venous phase, IC value was 37.65(100μg/ml); **(C)** Iodine map at delay phase, IC value was 38.94 (100μg/ml); **(D)** The histopathology (HE, magnification: ×200) showed adenocarcinomas cells infiltrate into lymphovascular structure (arrow).

**Figure 3:**
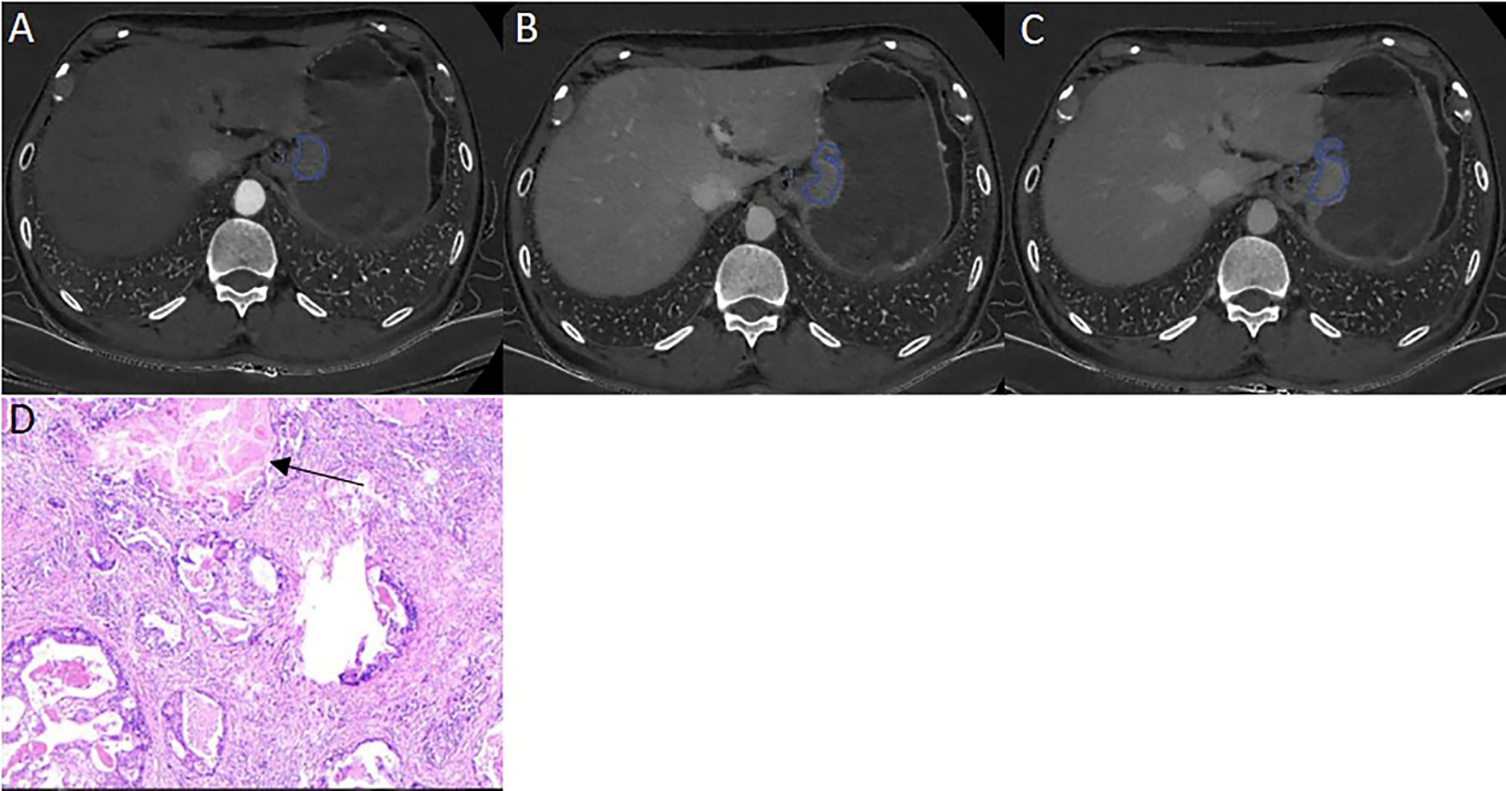
A 46 years old male patient with pathologically confirmed gastric adenocarcinoma, staging of pT3N0M0, LVI negative. The tumor thickness was 18.23 mm, Borrmann classification of III. **(A)** Iodine map at arterial phase, tumor was hyperintense, IC value was 21.34(100μg/ml); **(B)** Iodine map at venous phase, IC value was 37.65(100μg/ml); **(C)** Iodine map at delay phase, IC value was 33.40 (100μg/ml); **(D)** The histopathology (HE, magnification: ×200) showed normal lymphovascular structure (arrow).

The ICVP, ICDP, nICVP, nICDP in LVI positive cohort (**Figure 2**) were significantly higher than those in LVI negative cohort (**Figure 3**) in both primary and validation dataset (all *P*<0.05). Tumor thickness, clinical T staging, CT reported LN status, Borrmann classification in LVI positive group were statistically different from those in LVI negative group in the two cohorts (all *P*<0.05). The prevalence of Borrmann III-IV, cT3-4a, CT reported LN positive status of LVI positive group was 22/133(16.54%), 49/133(36.84%), 70/133(52.63%) in primary cohort, and was 17/62(27.42%), 49/62(79.03%), 43/62(69.35%) in validation cohort, respectively (**Table 3**), all were higher than LVI negative group.

**Table 3:** Comparison of CT parameters between LVI positive and negative group in the primary and validation datasets.

Parameters		Primary dataset				Validation dataset			
		LVI (-)(n = 91)	LVI (+)(n = 133)	*t*	*P*	LVI (-)(n = 50)	LVI (+)(n = 62)	*t*	*P*
Borrmann classification	I	59	15	6.462	<0.001*	19	10	10.710	0.004*
	II	37	45			27	35		
	III	2	19			4	17		
	IV	0	3			0	0		
Tumor thickness (mm)	Range: 5.3-38.2	13.48 ± 5.65	16.77 ± 6.01	5.345	<0.001*	15.49 ± 6.04	18.26 ± 5.12	2.627	0.010*
cT	1	44	7	6.014	<0.001*	7	1	19.971	<0.001*
	2	34	26			23	12		
	3	17	42			18	39		
	4a	3	7			2	10		
CT reported LN status	Negative	74	63	2.910	0.002*	35	19	17.169	<0.001*
	Positive	17	70			15	43		
ICAP (100μg/ml)		20.36 ± 6.90	17.83 ± 4.90	1.692	0.093	21.52 ± 7.76	21.07 ± .28	0.339	0.736
nICAP		0.18 ± 0.07	0.18 ± 0.06	1.833	0.068	0.20 ± 0.08	0.19 ± 0.05	0.818	0.415
ICVP(100μg/ml)		25.54 ± 7.19	25.35 ± 6.17	4.262	<0.001*	28.97 ± 7.43	30.62 ± 6.52	1.251	0.214
nICVP		0.44 ± 0.11	0.52 ± 0.13	4.240	<0.001*	0.49 ± 0.10	0.54 ± 0.10	2.552	0.012*
ICDP(100μg/ml)		21.10 ± 5.14	25.60 ± 6.67	4.993	<0.001*	26.89 ± 6.53	29.78 ± 6.49	2.343	0.021*
nICDP		0.52 ± 0.09	0.64 ± 0.15	6.332	<0.001*	0.56 ± 0.11	0.66 ± 0.12	4.326	<0.001*

AP, arterial phase; VP, venous phase; DP, delayed phase; HU, Hounsfield unit; IC, iodine concentration; nIC, normalized iodine concentration; LVI, perineural invasion; (-), negative; (+), positive; *P < 0.05.

### Development and validation of individualized predictive nomogram

**Figure 4:**
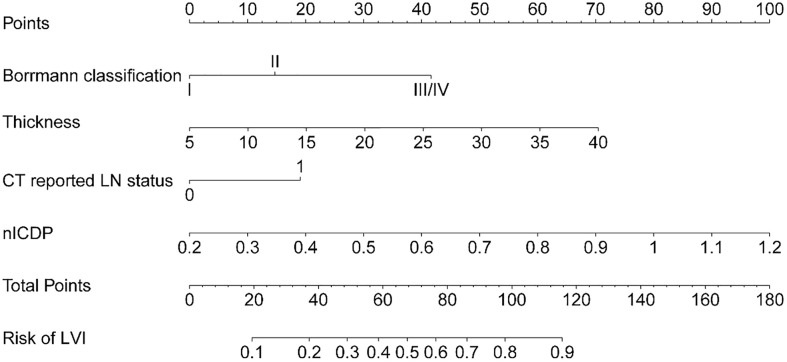
The developed nomogram, incorporating Borrmann classification, tumor thickness, CT reported LN status, and nICDP.

**Figure 5:**
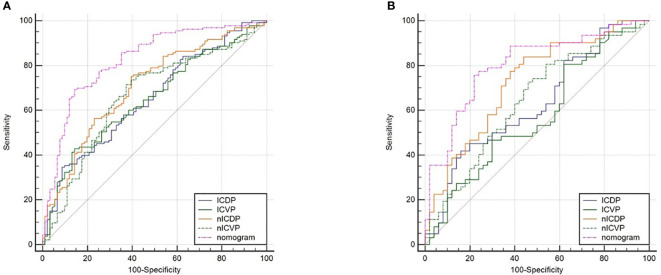
ROC analyses of IC parameters and the nomogram for the prediction of lymphovascular invasion in the primary dataset **(A)** and validation dataset **(B)**. The nomogram yielded the highest area under the curve of 0.825 (95%CI, 0.769-0.872) in the primary dataset and 0.802 (95%CI, 0.716-0.871) in the validation dataset.

When including significant preoperative parameters (tumour thickness, Borrmann classification, cT, CT reported LN status, ICVP, ICDP, nICVP, nICDP) in primary cohort into multivariable analysis, results revealed tumor thickness, Borrmann classification, CT reported LN status and nICDP were independent predictors for LVI (**Table 4**). Incorporating the above indicators, a nomogram was built to predict LVI probability individually (**Figure 4**). The nomogram had good performance for discrimination between LVI positive and negative with AUCs of 0.825(95% CI, 0.769-0.872) in the primary cohort and 0.802 (95%CI, 0.716-0.871)in the validation cohort (*Z*=11.295, 7.146, all *P*<0.001) (**Figure 5**; **Table 5**). Delong test showed the nomogram exhibited statistically higher AUC than ICVP, ICDP, ICVP, ICDP, respectively (*Z*=4.394, 4.594, 4.104, 3.713, *P*<0.001) in primary cohort, and in validation cohort (*Z*=4.031, 3.322, 3.134, 2.066, *P*=0.0001, 0.0009, 0.0027, 0.0389). There was no statistical difference of AUC yielded by the nomogram between primary and validation cohort (*Z*=0.891, *P*=0.173).

**Table 4:** Risk Factors for lymphovascular invasion in gastric cancer.

Variable	Nomogram			
	β	Wald	OR (95% CI)	*P*
Borrmann classification	0. 823	9.420	2.278 (1.347-3.854)	0.002
nICDP	4.561	9.937	95.640 (5.612-1629.828)	0.002
CT reported LN status	0.853	5.360	2.347 (1.140-4.832)	0.021
Thickness	0.091	8.630	1.095 (1.031-1.164)	0.003

IC, iodine concentration; DP, delayed phase; OR, odds ratio; CI, confident interval.

**Table 5:** ROC analyses of ICs and the developed nomogram.

Variable	Primary dataset						Validation dataset					
	AUC (95%CI)	Sensitivity	Specificity	Accuracy	*Z*	*P*	AUC (95%CI)	Sensitivity	Specificity	Accuracy	*Z*	*P*
ICVP(100μg/ml)	0.651(0.584-0.713)	0.429	0.857	0.643	4.102	<0.001	0.572 (0.475-0.665)	0.806	0.380	0.593	1.301	0.193
ICDP(100μg/ml)	0.653(0.586-0.715)	0.768	0.346	0.557	4.146	<0.001	0.627 (0.530-0.716)	0.419	0.840	0.623	2.373	0.018
nICVP	0.663(0.597-0.725)	0.737	0.604	0.671	4.353	<0.001	0.641 (0.545-0.730)	0.806	0.460	0.633	2.662	0.008
nICDP	0.705(0.641-0.764)	0.692	0.604	0.648	5.820	<0.001	0.7254 (0.632-0.805)	0.839	0.560	0.700	4.632	<0.001
Nomogram	0.825(0.769-0.872)	0.692	0.857	0.775	11.295	<0.001	0.802 (0.716-0.871)	0.758	0.780	0.769	7.146	<0.001

AP, arterial phase; VP, venous phase; DP, delayed phase; IC, iodine concentration; nIC, normalized iodine concentration.

### Calibration and clinical use of the nomogram

**Figure 6:**
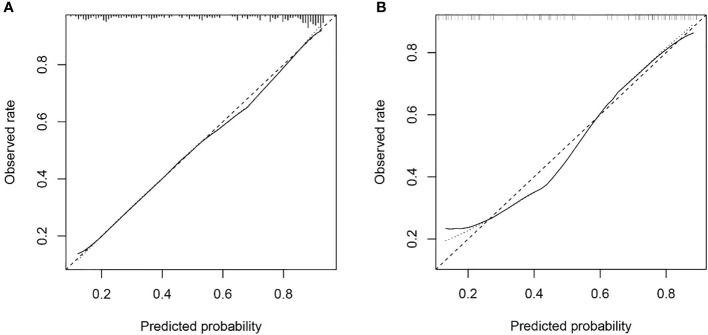
The calibration curve of nomogram in the primary dataset **(A)** and validation dataset **(B)** showed good agreement between the predicted probability of lymphovascular invasion (LVI) by the nomogram and actual probability of LVI after surgery. Calibration curves depict the calibration of each model in terms of the agreement between the predicted risks of LVI and observed rate of LVI. The y-axis represents the actual. The x-axis represents the predicted LVI prevalence. The diagonal dotted line represents a perfect prediction by an ideal model. The solid line represents the performance of the nomogram, of which a closer fit to the diagonal dotted line represents a better prediction.

**Figure 7:**
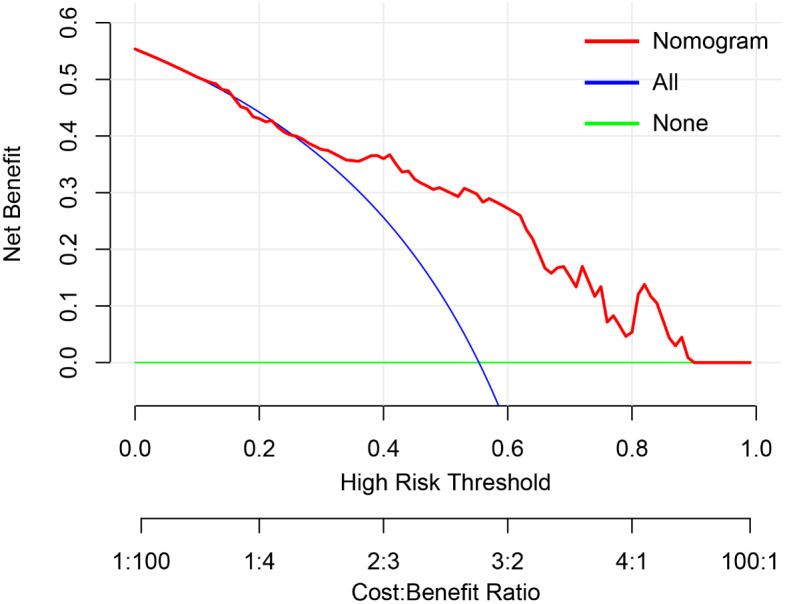
The decision curve analysis of nomogram in validation cohort. The y-axis measures the net benefit. The red line represents the developed nomogram. The blue line represents the assumption that all patients are presented with lymphovascular invasion (LVI). The green line represents the assumption that no patients are presented with LVI. The net benefit was calculated by subtracting the proportion of all patients who are false positive from the proportion who are true positive, weighting by the relative harm of forgoing treatment compared with the negative consequences of an unnecessary treatment. Here, the relative harm was calculated by. $\left(\frac{pt}{1-pt}\right)$ , “pt” (threshold probability) is where the expected benefit of treatment is equal to the expected benefit of avoiding treatment; at which time a patient will opt for treatment informs us of how a patient weighs the relative harms of false-positive results and false-negative results. $\frac{a-c}{b-d}$ ) = $\frac{1-pt}{pt}$ ); a−c is the harm from a false-negative result; b−d is the harm from a false positive result. a, b, c and d give, respectively, the value of true positive, false positive, false negative, and true negative (23) The nomogram exhibited higher net benefit in predicting LVI positive status across the range of threshold probabilities from 0.22 to 0.90 than the treat-none and treat-all strategy.

The Hosmer & Lemeshow test and calibration curve (**Figure 6**) showed good agreement between observed probability and the predicted probability by nomogram in the primary and validation dataset (*χ^2 =^* 8.337, 8.695, *P*=0.401, 0.369). The DCA of the nomogram in the validation cohort was demonstrated in **Figure 7**. The nomogram exhibited higher net benefit in differentiating LVI positive status from LVI negative status across the range of threshold probabilities from 0.22 to 0.90 than the treat-none and treat-all strategy.

## Discussion

This study presented a spectral CT quantification based nomogram for the preoperative individualized prediction of LVI with acceptable predictive performance. The nomogram incorporated three preoperatively available items of tumor thickness, CT reported LN status, Borrmann classification and nICDP. The nomogram was easy-to-use, quantitative and non-invasive, which could successfully stratify patients according to their risk of LVI.

Meng Y et al. (12) found clinical TNM stage was associated with LVI in GC. Chen X et al. (15) reported radiomics features related to tumor size and heterogeneity were top ranked indicators for predicting LVI, clinical T and N stage were independent risk factors for LVI. Our results of tumor size and CT reported LN status was predictive for LVI were consistent with the above studies. Besides, we found CT based Borrmann classification was significant in multivariable analysis and contained in model construction. Borrmann classification descripts tumor aggressiveness by tumor size, infiltration scale and the presence of ulceration, which represents distinct biological entities and reflect tumor aggressiveness (22). A preliminary work (21) proved Borrmann classification was an independent risk factor for LNM in GC, thus, we continued to analyze Borrmann classification in this study and found it is an independent predictor for LVI. Based on these findings, tumor thickness, CT reported LN status, and Borrmann classification were considered as easy-to-obtain risk factors of LVI in GC. To our knowledge, this is the first investigation on the relationship of spectral CT and LVI in GC. In terms of ICs, nICDP was screened as an independent risk factor for LVI in the present study, which added a quantitative imaging marker for operative prediction of LVI. Tumor thickness, CT reported LN status, Borrmann classification and nICDP were selected to build a predictive model for LVI in the primary datasets and validated in an independent external prospective dataset. Compared with Meng Y et al’s model, our model appears simple, quantitative and easy to use, it is superior to any other ICs with relatively high AUCs of 0.825 and 0.802 in primary and validation dataset. These findings support the selection of variables for model development is reasonable and feasible.

Several researchers have explored the association of multi-enhanced CT with LVI in GC, but with inconsistent results (24, 25). For example, Yin et al. (24) showed that CERAP (contrasted enhanced ratio at arterial phase) was significant for LVI, but Ma Z et al. (13) stated that Δpp (=CT attenuation at VP minus that at non-enhanced phase) was an independent predictor. It is noticeable that these studies were retrospective one centered study, the efficacy of enhanced CT for preoperative LVI assessment is far from clinical satisfactory. Vascular endothelial growth factor (VEGF) family can induce both angiogenesis and lymphangiogenesis (25), and tumor angiogenesis was highly related to LVI in GC. ICs showed perfect consistency with true iodine deposition in tube experiment (17). ICVP and nICVP were proved to be positive correlation with microvascular density (MVD) and VEGF on gross specimen of GC after surgery (18), which means that ICs can reflect tumor angiogenesis quantitatively and non-invasively. Our results showed ICVP, ICDP, nICVP and nICDP in LVI positive group was statistically higher than those in LVI negative group in both datasets, a significant finding suggested that IC values enable effective discrimination between different LVI status in GC. Gastric adenocarcinoma is well-known tumor with abundant fibrosis and featured by persistent enhancement after contrast agent administration (16, 17). We prospectively applied bolus tracking technique to set individualized acquisition timing, and the DP was obtained around 90s delay. We observed that although CT attenuation at DP decreased mildly, primary tumor still presented relatively high enhancement. Theoretically, DP enhancement at 90s reflects the fibrosis abundance nature of GC, ICDP represents the late-phase retention of contrast agent in interstitial spaces. LVI is refer to destruction of lymphovascular structures by tumor cell infiltration (9, 10), may increase the microvascular permeability and locoregional tumor cell density, and account for higher ICs and nICs at venous phase and delay phase.

In previous studies, nICs was introduced to minimize or eliminate circulation varies among individuals and exhibited comparable performance in tumor characteristic description, staging and treatment response evaluation (15–17), especially nICVP. We found that nICDP was significant in multivariable analysis and predictive for LVI, rather than nICVP. Differences may mainly due to different protocol and timing, the previous studies used dual phase enhanced protocol, the VP was acquired at 60~70s after contrast agent administration, whereas we applied triple phase enhanced method, DP was obtained around 90s delay, when primary GC still present with persistent enhancement, nICDP at this time point represent the balance of blood supply and the late-phase retention of contrast agent in interstitial spaces, which is in accordance to the abundant fibrosis nature of GC. Despite nICs were useful and relatively reliable, the usage of nICs has not achieved worldwide consensus and generalization. More studies are needed to verify the predictive value of nICs in GC.

Several researchers developed nomograms for LVI prediction with acceptable AUCs. Meng Y et al. (12) proposed a nomogram consisted of clinical indicators with AUC of 0.774 in the testing datasets. Chen X et al. (15) and Li Q et al. (14) proposed radiomics models from enhanced CT images and yielded AUC of 0.792 and 0.725 in testing dataset respectively. Different from the existing nomograms, our nomogram firstly contained quantitative imaging marker (nICDP) and clinically meaningful and available features (CT reported LN status, tumor thickness and Borrmann classification) from one stop scan on spectral CT with comparable or better AUC of 0.802 in the validation dataset, but without complicated radiomics algorithm. The predictive efficacy of the nomoram was externally validated in a prospective cohort, suggestive of its good generalization.

Study limitations include that data acquired on fast kV DECT platform, which may not be applicable to other DECT platforms. Besides, laboratory and genetic markers have not yet been incorporated in the nomogram. Therefore, multiscale studies are expectable to establish a more comprehensive method to predict LVI in patients with GC.

In conclusion, this study presents and externally validates a spectral CT based and clinically available predictive tool that combined quantitative parameter of nICDP and significant risk factors for preoperative LVI in GC with favorable accuracy.

## Data availability statement

The raw data supporting the conclusions of this article will be made available by the authors, without undue reservation.

## Ethics statement

The studies involving human participants were reviewed and approved by the Affiliated Cancer Hospital of Zhengzhou University (Henan Cancer Hospital) and the first Affiliated Hospital of Zhengzhou University. The written informed consent was obtained from each patients in the Affiliated Cancer Hospital of Zhengzhou University (Henan Cancer Hospital) and waived in the first Affiliated Hospital of Zhengzhou University.

## Author contributions

Guarantor of integrity of the entire study: JL and J-rQ. Study conception and design: J-rQ and J-bG. Data acquisition: JL and RW. Pathologic diagnoses: YW. Imaging analysis: JL and RW. Statistical analyses: JL and J-rQ. Manuscript writing: JL. Manuscript editing: J-rQ and J-bG. All authors contributed to the article and approved the submitted version.

## Funding

Science and Technology Development Foundation of Henan Province (202102310736), Henan Provincial Medical Science and Technology Project (SBGJ202003011), Projects of the General Programs of the National Natural Science Foundation of China (No.81972802, 82202146), Special funding of Henan Health Science and Technology Innovation Talent Project (No.YXKC2020011, No. YXKC2021054).

## Conflict of interest

The authors declare that the research was conducted in the absence of any commercial or financial relationships that could be construed as a potential conflict of interest.

## Publisher’s note

All claims expressed in this article are solely those of the authors and do not necessarily represent those of their affiliated organizations, or those of the publisher, the editors and the reviewers. Any product that may be evaluated in this article, or claim that may be made by its manufacturer, is not guaranteed or endorsed by the publisher.
